# Single Cell Analysis of Lung Lymphatic Endothelial Cells and Lymphatic Responses during Influenza Infection

**DOI:** 10.35534/jrbtm.2024.10003

**Published:** 2024-02-19

**Authors:** Jian Ge, Hongxia Shao, Hongxu Ding, Yuefeng Huang, Xuebing Wu, Jie Sun, Jianwen Que

**Affiliations:** 1 Columbia Center for Human Development & Division of Digestive and Liver Diseases, Department of Medicine, Columbia University Irving Medical Center, New York, NY 10032, USA; 2 Haihe Hospital, Tianjin University, Tianjin 300350, China; 3 Department of Pharmacy Practice and Science, College of Pharmacy, University of Arizona, Tucson, AZ 85724 USA; 4 Department of Microbiology & Immunology, Columbia University Irving Medical Center, New York, NY 10032, USA; 5 Department of Medicine, Department of Systems Biology, Columbia University Irving Medical Center, New York, NY 10032, USA; 6 Carter Immunology Center, University of Virginia, Charlottesville, VA 22908, USA

**Keywords:** Lymphatic endothelial cells (LECs), Single cell, Influenza infection, lung injury, Regeneration, scRNA-seq, PD-L1

## Abstract

Tissue lymphatic vessels network plays critical roles in immune surveillance and tissue homeostasis in response to pathogen invasion, but how lymphatic system *per se* is remolded during infection is less understood. Here, we observed that influenza infection induces a significant increase of lymphatic vessel numbers in the lung, accompanied with extensive proliferation of lymphatic endothelial cells (LECs). Single-cell RNA sequencing illustrated the heterogeneity of LECs, identifying a novel PD-L1^+^ subpopulation that is present during viral infection but not at steady state. Specific deletion of *Pd-l1* in LECs elevated the expansion of lymphatic vessel numbers during viral infection. Together these findings elucidate a dramatic expansion of lung lymphatic network in response to viral infection, and reveal a PD-L1^+^ LEC subpopulation that potentially modulates lymphatic vessel remolding.

## 1. Introduction

Lymphatic vessels are integral components of the respiratory system, playing a vital role in maintaining pulmonary homeostasis and fluid balance. These vessels, characterized by their unique anatomy and function, are crucial in immune surveillance and the transport of interstitial fluids [1–3]. Lymphatics in the lung are particularly important for draining excess fluid, a function essential for efficient gas exchange and preventing pulmonary edema during infection and injury [4,5]. In consistency with their function, the architecture of lymphatic vessels in the lung is distinct, consisting primarily of lymphatic endothelial cells (LECs) and lacking the surrounding smooth muscle cells (Reed et al. 2019). LECs are also vital for lung development, especially fluid clearance, and lung inflation during the transition from prenatal to postnatal life [6]. Deletion of the master regulator *Prox1* leads to failed commitment of LECs [7–9].

Although lung regeneration post infection has been extensively studied [10–14], the critical role of lymphatics in pulmonary health remains poorly understood. Notably, the numbers of lymphatic vessels are increased following lung injuries caused by bleomycin or *Mycoplasma pulmonis* infection [15,16], indicating a reactive adaptation of lymphatic vessels. However, the mechanisms behind this adaptation, particularly in the context of influenza infection, have not been fully elucidated. Single-cell RNA sequencing has been instrumental for our understanding of population dynamics during infections by virus like SARS-CoV-2 and influenza [17,18]. However, given that LEC is a small cell population in the lungs [19], previous studies often analyze the population along with endothelial cells [20]. More recently, embryonic and neonatal (P0) LECs were purified for scRNA-seq analysis, leading to the revelation of the important role for the transcription factor *c-JUN* in the opening of lung lymphatic vessels at birth [21]. Although LECs among other cell populations were reported to be altered during influenza infection [17], comprehensive analysis of LECs is lacking. In this study we used the *Prox1-CreER* mouse line to specifically characterize the dynamic changes of the lymphatic vessels during influenza infection. Our findings delineate a distinct response of LECs to influenza infection, characterized initially by vessel dilation and subsequently by an increase in vessel numbers. scRNA-seq analysis of purified LECs further uncovered the presence of heterogenous LEC populations during infection. Our subsequent study of a *Pd-l1*^+^ subpopulation suggests that *Pd-l1* is involved in the regulation of lymphatic vessel numbers upon influenza challenge.

## 2. Material and Methods

### 2.1. Mouse

*Prox1-CreERT2* (Jackson Laboratories, Strain #: 022075; Bar Harbor, ME, USA) and *R26R*^*tdTomato*^ mice (Jackson Laboratories, Stock #: 007905) were crossed to generate *Prox1-CreERT2; R26R*^*tdt*^ mice, *Prox1-CreERT2; R26R*^*tdT*^ mice at 8 weeks were i.p. injected within tamoxifen (100 mg/kg) to lineage label the lymphatic endothelial cells.

*Prox1-CreERT2; R26R*^*tdT*^ mice were further bred with *Pd-l1*^*loxp/loxp*^ mice (Jackson Laboratories, Strain #:036255) which have loxP sites flanking exons 2–3 of the *Cd274* gene.

All animal experiments were approved by the Institutional Animal Care and Use Committee (IACUC) at Columbia University. Mice were intranasally infected with PR8 (40 μL) at a dose of 150 PFU per mouse under tribromoethanol anesthesia. All infected mice were monitored, body weight was measured including initial timepoint.

### 2.2. Tissue Clearing and Light-sheet Microscopy

*Prox1-CreERT2; R26R*^*tdT*^ mice were euthanized, and perfusion was conducted using a 23G needle with PBS followed by 4% PFA. Tissues were fixed in 4% PFA, embedded in a hydrogel mixture, and polymerized. 8% SDS was used for delipidation of samples via passive incubation. Heme removal was achieved using a 25% Quadrol solution. Refractive Index Matching (RIM) was performed using Histodenz solutions with stepwise increments in refractive indices for optimal imaging clarity. The tissue was imaged by light Sheet Microscope (Leica SP8-DLS; Leica, Mannheim, Germany).

### 2.3. Immunofluorescence Staining and Confocal Image Microscopy

Immunofluorescence staining was performed as previously described [22,23]. Antibodies used in this study included Rat anti-Ki67 (Invitrogen, 14-5698-82; Carlsbad, USA), Goat anti tdTomato (biorbyt, orb182397; Durham, NC, USA), Rabbit anti-PROX1 (Abcam, ab101851; Cambridge, UK), and Goat anti Flt-4/VEGFR3 (R&D systems, AF743-SP; Minneapolis, MN, USA). Immunostained slides were imaged by Zeiss LSM 710 Confocal Microscope or Leica Stellaris 8 Confocal Microscope.

### 2.4. Lung Function Assay

Pulmonary function parameters in mice, such as tidal volume, were measured using the Buxco FinePointe NAM 2-Site Station (DSI, Serial Number: 844578). Mice were carefully placed in the device’s chamber, leaving the mice for 5 min, then their respiratory patterns including tidal volume were recorded for 25 min (>700 records per measurement). In our experiments, we employ tidal volume values for the direct comparison between animal groups. However, due to variations in technique, these absolute values may differ when measured by others. Data was performed ANOVA and Tukey’s HSD test analysis in Python.

### 2.5. Lymphatic Drainage Assay

50 μL of 5 mg/mL dextran-FITC (10,000 kDa, Thermo Fisher Scientific, D7171; Waltham, MA, USA) was instilled intratracheally in anesthetized mice, Evans blue was i.p. injected 20min prior to sac the mice to label the lung drainage lymph nodes, then mice were euthanized after 50 min. Mice were perfused by PBS, lung and lung drainage lymph nodes were harvested for cryosection, and tissue sections were imaged using a fluorescence microscope (Zeiss LSM 710 Confocal Microscope; Carl Zeiss, Jena, Germany) to analyze dextran signal intensity.

### 2.6. Single-cell RNA-sequencing Analysis

Mice were sacrificed and perfused through injection with PBS into the right ventricle, and lungs were inflated with 1 mL 1 mg/mL collagenase and disease (Sigma-Aldrich, 10269638001, Burlington, VT, USA) dissolved in DMEM medium via the trachea. Lungs were then minced into small pieces with a razor blade and incubated at 37 °C for 45 min within 10 mL 1 mg/mL collagenase and dispase. The minced tissues were dissociated further with the long cannule needle, and the resulting cell suspension was filtered through 70 μm filter. Red blood cells were removed with Red Blood Cell Lysing Buffer Hybri-Max^™^ (Sigma-Aldrich, R7757). TdTomato positive and CD45 negative cells were FACS sorted for 10× Genomics Single Cell RNA sequencing. Single-cell RNA sequencing data were processed using the Seurat R package. After initial quality control, cells were filtered, normalized, and variable features identified. Dimensionality reduction was performed using PCA and UMAP. Clusters were identified and characterized by differential gene expression analysis, and findings were visualized through various plots. Gene Ontology enrichment analyses provided further biological insights.

### 2.7. Quantification and Statistical Analysis

All data are generated by GraphPad Prism in the means ± SEM. Unpaired two-tailed Student’s *t*-test was calculated to determine statistical significance. *p* < 0.05 or less were considered statistically significant. For the quantification of the number and size of lymphatic vessels, dextran intensity, Ki67+ ratio and lung injury area ratio, at least three animals were included for each group. For the quantification of body-weight change, a two-way repeated measures ANOVA was conducted, indicating no significant main effect between the control and *Pd-l1*^ΔLEC^ mice.

## 3. Results

### 3.1. Influenza Infection Causes Lymphatic Vessel Dilation Followed by an Increase in Vessel Numbers

**Figure 1.**
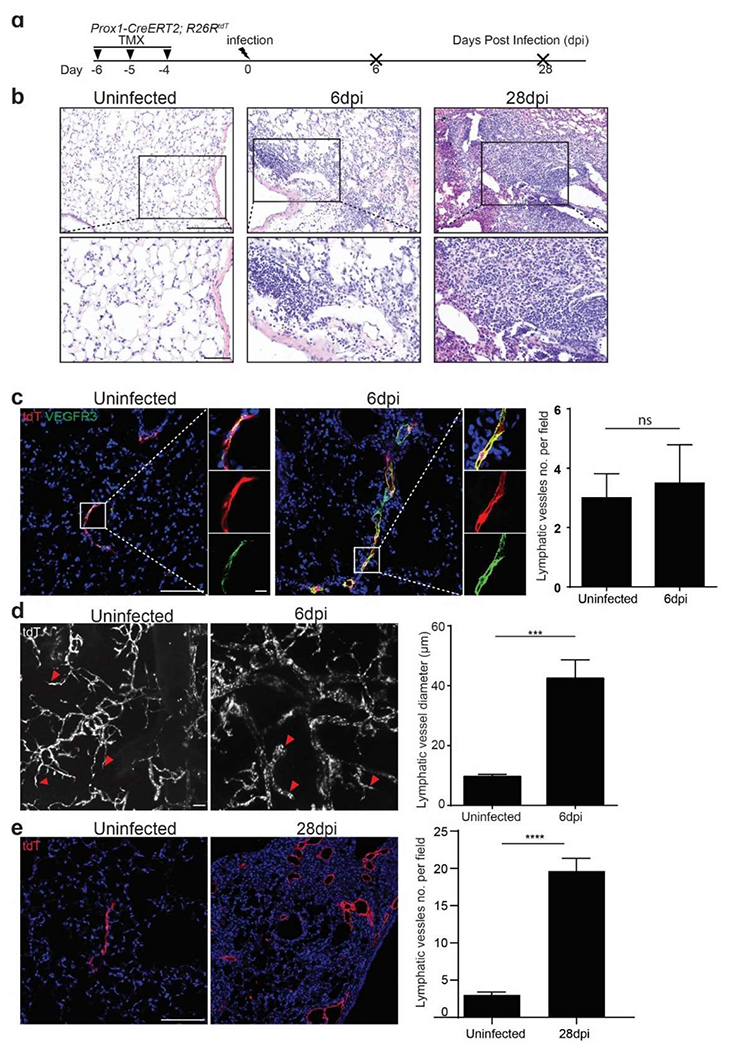
Influenza infection induces lymphatic vessel dilation followed with an increase in number. (**a**) Experimental timeline indicating the administration of tamoxifen (TMX), influenza infection and the days post-infection (dpi). (**b**) Representative hematoxylin and eosin (H&E) staining of lung sections from control and infected *Prox1-CreERT2; R26R*^*tdT*^ mice at 6 and 28 dpi. (**c**) Immunofluorescence staining for VEGFR3 (green) and tdTomato (red) of lung sections from control and virus-infected mice. Note nuclei counterstained with DAPI (blue). Quantification of lymphatic vessels per field is shown on the right (mean± SEM. *n* = 4. n.s., not significant). (**d**) Light-sheet microscopy images of tdTomato expression in control and virus-infected lung tissues at 6 dpi. Quantification of the lymphatic vessel diameter is shown in the adjacent graph (mean± SEM, *n* = 3 *** *p* < 0.001). (**e**) Immunofluorescence of tdTomato in the virus-infected lung sections at 28 dpi. Quantification of lymphatic vessels is shown on the right (mean± SEM, *n* = 4 for uninfected, *n* = 11 for 28 dpi, **** *p* < 0.0001). Scale bars: 200 μm for **b** (60 μm for insets), 100 μm for **c–e** (10 μm for insets in **c**).

To study the response of lymphatic vessels to influenza infection, we administrated three doses of tamoxifen into *Prox1-CreERT2; R26R*^*tdT*^ mice to label the LECs in the lungs, and then we challenged the mice with H1N1 influenza PR8 (Figure 1a). We harvested the lungs at 6 and 28 days post-infection (dpi) (Figure 1b). The lung structures were minimally changed but with increased accumulation of immune cells in the injured area at 6 dpi. In contrast, the histological changes were apparent, and the numbers of immune cells were dramatically increased in the injured area when examined at 28 dpi (Figure 1b). We then used VEGFR3 as a marker for LECs, which were also positive for tdTomato (tdT), to visualize the lymphatic vessels via confocal microscopy. At the early stage of infection (6 dpi) the number of lymphatic vessels was not dramatically altered (Figure 1c), while we observed a 4-fold increase in the diameters of lymphatic vessels using tissue clearing and 3D imaging of the whole lung by light-sheet microscopy (Figure 1d). By 28 dpi the number of lymphatic vessels was increased by 6 folds (Figure 1e), indicating a significant expansion of the lymphatic network. Together our findings demonstrated that the lymphatic vessels underwent initial dilation followed by dramatic increases in the number of lymphatic vessels. The dilation can be seen as a functional adaptation in response to viral infection, allowing for two purposes: (1) return of excess interstitial fluid to the blood to maintain tissue fluid balance; (2) transport inflammatory cells to facilitate the resolution of inflammation.

### 3.2. Influenza Infection Induces Transient But Intensive Proliferation of LECs Accompanied with a Change in Lymphatic Function

**Figure 2.**
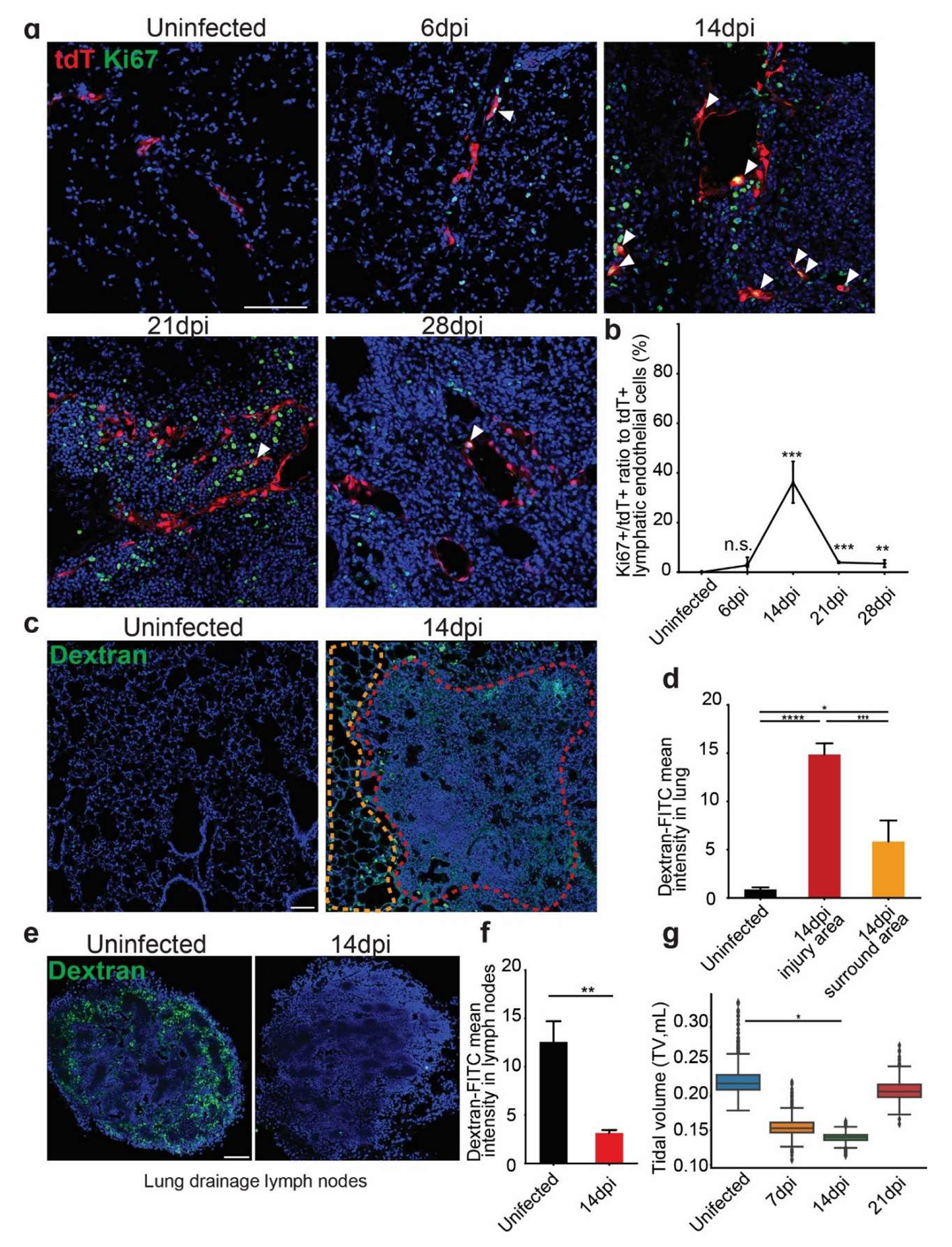
Influenza infection induces transient but intensive proliferation of LECs accompanied with a change in lymphatic function. (**a**) Immunofluorescence staining for Ki67 (green) in tdTomato-labeled lymphatic endothelial cells (LECs) (red) in the lung at different time points post viral infection. White arrowheads indicate Ki67^+^tdTomato^+^ proliferating lymphatic endothelial cells. (**b**) Quantification of the percentage of Ki67^+^ cells in tdTomato^+^ LECs. Note proliferation peaks at 14 dpi (mean ± SEM, *n* = 4 for uninfected/6 dpi/14 dpi, *n* = 3 for 21 dpi/28 dpi, ***p < 0.001, **p < 0.01; n.s., not significant). (**c** & **e**) Representative images of dextran-FITC drainage in lungs and lung drainage lymph nodes. (**d** & **f**) Quantitation of FITC-dextran mean intensity (mean ± SEM, *n* = 3, ****p < 0.0001, ***p < 0.001, **p < 0.01, *p < 0.05). (**g**) Tidal volume (TV) at different time points post infection (*p < 0.05). Scale bars: 100 μm for **a** and **c**.

Since we observed increased numbers of lymphatic vessels, we used the proliferation marker Ki67 to co-stain with tdT (Figure 2a). There were very few proliferative LECs at 6 dpi, consistent with early phenotype that the lymphatic vessels dilation without increasing in numbers. The temporal profile revealed a stark increase in Ki67^+^tdT^+^ cells, indicating intensive proliferation of LECs (35%) at 14 dpi, and by 28 dpi the numbers of Ki67^+^tdT^+^ cells diminished (Figure 2b). These findings confirmed a transient proliferative response during influenza infection.

We next used dextran assays to assess whether the increased numbers of lymphatic vessels are associated with any changes in drainage, an important function of the lymphatic system (Figure 2c). Intratracheal instillation of FITC-labeled Dextran was tested for uptake to the lymphatics from the interstitial. A marked increase in FITC-dextran intensity within the injured areas illustrated the compromised lymphatic drainage function (Figure 2d). Notably, the FITC-dextran intensity in the injured area was significantly higher than the intensity in the surrounding uninjured areas, highlighting the localized impact of infection on lymphatic function. Consistently, the intensity is impaired in lung drainage lymph nodes after influenza infection (Figure 2e,f). In addition, lung function was also impacted by the infection as we observed a gradual reduction of tidal volume, associated with the dysfunction of lymphatics at around 14 dpi (Figure 2g).

### 3.3. Single-cell Transcriptomic Analysis Identifies a PD-L1^+^ LEC Subpopulation during Influenza Infection

**Figure 3.**
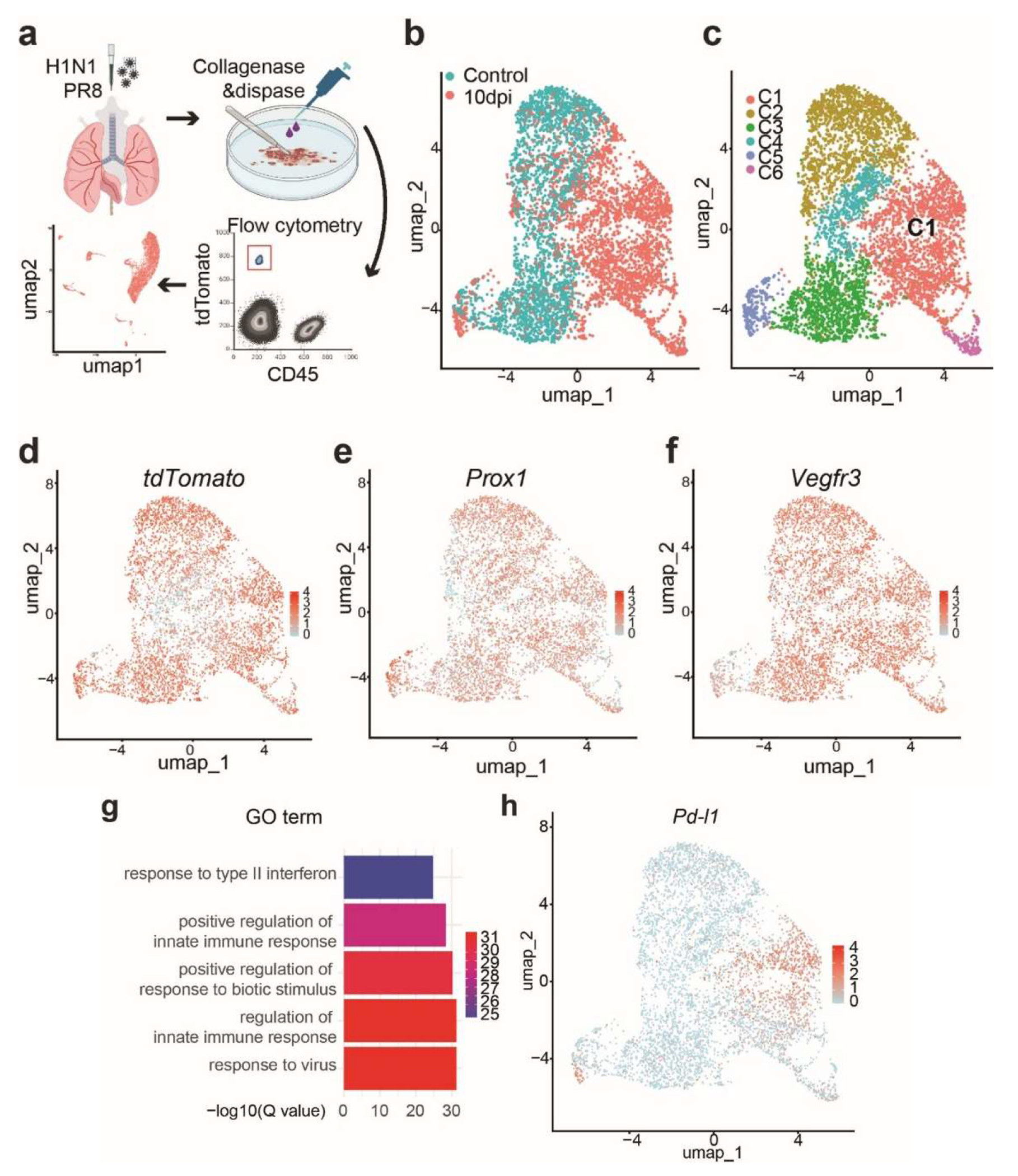
scRNA-Seq analysis identifies a PD-L1^+^ LEC subpopulation during influenza infection. (**a**) scRNA-seq experimental diagram. (**b**) UMAP clustering of single-cell transcriptomes of LECs purified from control (*Prox1-CreERT2; R26R*^*tdT*^ uninfected) and virus-infected lungs. Note lung tissues were collected from *Prox1-CreERT2; R26R*^*tdT*^ mice at 10 dpi. (**c**) Identification of 6 distinct LEC clusters (C1–C6) based on gene expression profiles. (**d**, **e** and **f**) Expression of *tdTomato, Prox1* and *Vegfr3* in distinct LEC clusters. (**g**) Gene Ontology (GO) analysis of signaling pathways enriched in cluster C1. −log10 (Q value) indicates the significance of enrichment. (**h**) The distribution of *Pd-l1* expression across LEC populations.

We next employed scRNA-seq analysis to determine the heterogeneity of LECs both at steady state and after influenza infection. We collected tdT^+^ CD45^−^ cells from the lungs of *Prox1-CreERT2; R26R*^*tdT*^ mice at 10 dpi or without infection (Figure 3a). In total 6,661 cells were subjected to scRNA-Seq. The Uniform Manifold Approximation and Projection (UMAP) plots displayed distinct populations of LECs (Figure 3b) and revealed 6 clusters (C1-C6) of LEC subpopulations when combining virus-infected and uninfected lungs (Figure 3c). Each of these cluster exhibited a distinct transcriptome profile (Table 1). As expected, all the isolated tdTomato^+^CD45^−^ sequenced cells (Figure 3d) were positive for *Prox1* and *Vegfr3* (Figure 3e,f), confirming their LEC identity. Interestingly, the C1 cluster was a unique subpopulation that was exclusively present in the influenza-infected lungs (Figure 3c). This cluster expressed Guanylate-binding protein family genes (*Gbps*) and Programmed death-ligand 1 (*Pd-l1*) (Figure 3h), which have been shown to mediate immune responses [24–26]. Cluster 3 LECs were characterized by the expression of Transforming growth factor beta induced (*Tgfbi*) and Proline and arginine rich end leucine rich repeat protein (*Prelp*), which are involved in the production of extracellular matrix-related proteins [27–29]. Cluster 6 LECs were positive for Ki67, and also expressed Spindle and kinetochore associated complex subunit 1 (*Ska1*) and Kinesin family member 18b (*Kif18b*), which are important for cell cycle progression and cell division [30–32].

Consistently, the gene ontology (GO) term enrichment analysis revealed that the C1 subpopulation of LECs were associated with immune response-related processes (Figure 3g), indicating a potential role in the host defense. Expression of the immune checkpoint molecule *Pd-l1* in this unique LEC subpopulation implied that they are involved in immunomodulatory actions during infection.

### 3.4. Pd-l1 Deletion in LECs Was Associated with an Increased Number of Lymphatics during Infection

**Figure 4.**
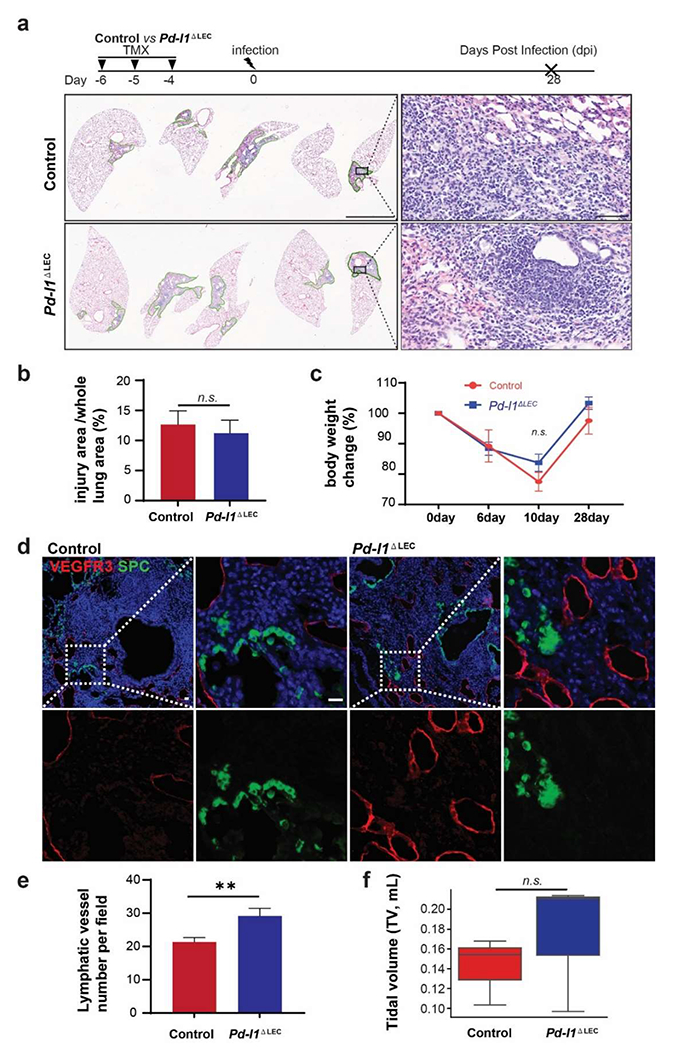
Pd-l1 conditional deletion leads to increased lymphatic vessels following viral infection. (**a**) H&E staining of lung sections from control (Prox*1-Cre-ER; Pd-l1*^*loxp/+*^) and *Pd-l1*^ΔLEC^ mice at 28 dpi. (b) Quantification injury areas across the whole lungs between control and *Pd-l1*^ΔLEC^ mice (mean ± SEM, *n* = 5 for control, *n* = 3 for *Pd-l1*^ΔLEC^, n.s.). (**c**) Body weight change of the mice following viral infection (mean ± SEM, *n* = 3 for control, *n* = 5 for *Pd-l1*^ΔLEC^, p=0.322). (**d**) Immunofluorescence staining for VEGFR3 (red) and SPC (green) in the lungs at 28 dpi. SPC was used to determine alveolar regeneration. (**e**) Quantification of lymphatic vessels per field (0.339 mm^2^) (mean ± SEM, *n* = 28 for control, *n* = 25 for *Pd-l1*^ΔLEC^, **p < 0.01). (**f**) Tidal volumes at 14 dpi (*p* = 0.7). Scale bars: 3 mm for **a** (60 μm for insets) and 20 μm for **d**.

We then tested the function of PD-L1 in LECs following viral infection. We generated Prox*1-Cre-ER; Pd-l1*^*loxp/loxp*^ mutants to specifically delete *Pd-l1* in LECs (*Pd-l1*^ΔLEC^). We did not observe a significant difference in histology and injured area between control and *Pd-l1*^ΔLEC^ mice at 28 dpi (Figure 4a,b). Consistently, mutant mice showed a similar pattern of post-injury weight recovery to controls (Figure 4c). These results suggest that overall systemic health and lung tissue recovery are not altered by the specific deletion of *Pd-l1* in LECs.

We then focused on the lymphatic vessels. *Pd-l1* deletion in LECs caused an increase in number of lymphatic vessels and an expansion of lymphatic network at 28 dpi compared to controls (Figure 4d), suggesting that PD-L1 suppress LEC differentiation and/or proliferation. There was also a trend that the tidal volume in *Pd-l1*^ΔEC^ mice was higher than in controls, although lack of statistic power (Figure 4f). Together, these results suggest that PD-L1 plays a negatively regulatory role in lymphatic remodeling in the lung during viral infection.

## 4. Discussion

Lymphatic vessels are important for immune actions and absorption of fluid from the interstitial space in response to tissue damage. Here, we showed the dilation of lymphatic vessels at the early stage of viral infection followed by an increase in the number of the vessels. The expansion of lymphatic vessels was associated with the presence of extensive proliferation of LECs. Our further analysis of LECs with scRNA-seq revealed 6 subpopulations, among which one subpopulation was induced by viral infection with the exclusive expression of *Pd-l1*. Although *Pd-l1* deletion had minimal impact on injury/repair after viral infection, the number of lymphatic vessels was increased in the *Pd-l1*^ΔLEC^ lungs.

The initial response to viral infection was the dilation of lymphatic vessels. This is a possible initial functional adaptation of the system, to maintain tissue fluid balance and allow inflammatory immune cells to egress for inflammation resolution [33]. The expansion of lymphatic vessel networks was accompanied by the increased proliferation of LECs which seemed to peak at around 14 dpi. Notably, although the number of lymphatic vessels was increased, the lung function seemed not to improve. It’s noted that lymphatic vessel proliferation may be associated with the progression of pathological states, like renal interstitial fibrosis [34], and fibrotic lung diseases including Chronic Obstructive Pulmonary Disease (COPD) [35,36]. Thus, the expanded lymphatic networks do not necessarily contribute to improved organ function. This can be complicated in patients undergoing lung transplantation, where the lymphatic system is not connected with the host. If additional viral infection occurs, the dysfunction lymphatic system can cause serious issues. On the other hand, LECs have been shown to serve as niche cells for the intestinal stem cells during injury/repair [37]. These lymphatic cells serve as an important source for *R-spondin 3* and *Wnt2*, promoting the expansion of intestinal stem/progenitor cells. Notably, *R-spondin 3* is upregulated in almost every LEC subpopulation based on our scRNA-seq data. However, our analysis demonstrated the number of SPC+ AT2 cells seemed unchanged following *Pd-l1* deletion in virus-infected *Pd-l1*^ΔLEC^ lungs. It will be interesting to determine whether/how exactly these LECs are involved in epithelial regeneration after viral infection in the future.

Our scRNA-seq analysis suggested heterogenous LECs following viral infection. In particular, a unique cluster of LECs were present in virus-infected lungs, expressing PD-L1 which is encoded by *Cd274* gene [38,39]. Interestingly, during viral infection the level of *Pd-l1* is increased in LECs in response to type 1 IFN, inhibiting LEC expansion while promoting LEC survival within the lymph nodes [40]. The upregulation of PD-L1 in the infected lungs could be part of a feedback mechanism to regulate the immune response. Our findings are in line with the previous studies showing that PD-1/PD-L1 interactions alter the function of T cells during respiratory viral infections [41–43]. PD-L1 has been shown to regulate invasiveness of lung fibrosis and genetic deletion or pharmacologically inhibition of PD-L1 reduces fibroblast invasion [44]. PD-L1 is a key player in tumor immune suppression, and studies have shown that PD-L1 signaling including PD-1/PD-L1 and CD80/PD-L1 is crucial for T cell migration, modulating transendothelial movements of Tregs and CD4 effector T cells [45]. Furthermore, *Pd-l1* expression in LECs has been shown to contribute to an immunosuppressive tumor microenvironment by facilitating regulatory T-cell expansion [46] and suppressing tumor-specific immunity, notably by inducing apoptosis in CD8^+^ central memory T cells in tumor-draining lymph nodes [47]. Here, we showed that *Pd-l1* expression was increased in LECs upon injury, and *Pd-l1* knockout mice exhibited increased lymphatic vessel numbers. It will be interesting to determine whether *Pd-l1* in LECs plays similar roles in modulation of immune cells following viral infection.

In conclusion, our findings highlight the dynamics of the lymphatic system in response to viral infection. The heterogeneity of LECs during homeostasis and injury/repair suggests distinct function of individual LEC function which warrants further study.

## Figures and Tables

**Table 1.** top 15 up-regulated genes in each cluster.

Gene Rank	Cluster 1	Cluster 2	Cluster 3	Cluster 4	Cluster 5	Cluster 6
**1**	*Serpina3f*	*Gm525*	*Gucy1a1*	*Cacna1e*	*Ppargc1a*	*Ska1*
**2**	*Rsad2*	*Chchd10*	*Ptn*	*Wdr17*	*Sbspon*	*Hist1h1b*
**3**	*Oasl2*	*Itih5*	*Tgfbi*	*Gm15261*	*Spp1*	*Hist1h2af*
**4**	*Ifi211*	*Npnt*	*Comp*	*Gm12353*	*Co16a5*	*Hist1h3b*
**5**	*Phf11b*	*Cd36*	*Cd300lg*	*Lysmd4*	*Irf6*	*Kif18b*
**6**	*Gbp5*	*Aqp1*	*Fxyd1*	*Stab2*	*Ephx2*	*Depdc1a*
**7**	*Gbp4*	*Hapln1*	*Rbp7*	*Sned1*	*Fibin*	*Hist1h2ae*
**8**	*Gbp2*	*Sparc*	*Fmo1*	*Lncpint*	*Lypd6*	*Nuf2*
**9**	*Sh2d5*	*Dipk1b*	*Gm1673*	*Arhgap6*	*Tmem45a*	*Hist1h2ag*
**10**	*Gbp6*	*Osr1*	*Slc8a1*	*Magi1*	*Ttll6*	*Pbk*
**11**	*Irf7*	*Pgf*	*Mboat1*	*Etl4*	*Mtcl1*	*Hist1h2ai*
**12**	*Pd-l1*	*Olfml2a*	*Tppp3*	*Adam12*	*Tox*	*Hist1h2ai*
**13**	*Gm4951*	*Selenop*	*Prelp*	*Rora*	*Pkp3*	*Shcbp1*
**14**	*Batf2*	*Trf*	*Pdlim3*	*Kansl1l*	*Ahsg*	*Hist1h3c*
**15**	*Gpx3*	*Ifitm10*	*Chp2*	*Cped1*	*Hgf*	*Nek2*

* *p*-adj value for all the listed genes is smaller than 5.6 × 10^−5^.

## Data Availability

GSE266192 Single Cell Analysis of Lung Lymphatic Endothelial Cells and Lymphatic Responses during Influenza Infection.
